# Mobilization‐based transplantation of young‐donor hematopoietic stem cells extends lifespan in mice

**DOI:** 10.1111/acel.13110

**Published:** 2020-02-03

**Authors:** Michael J. Guderyon, Cang Chen, Anindita Bhattacharjee, Guo Ge, Roman A. Fernandez, Jonathan A. L. Gelfond, Karla M. Gorena, Catherine J. Cheng, Yang Li, James F. Nelson, Randy J. Strong, Peter J. Hornsby, Robert A. Clark, Senlin Li

**Affiliations:** ^1^ Department of Medicine UT Health San Antonio San Antonio TX USA; ^2^ Department of Epidemiology and Biostatistics UT Health San Antonio San Antonio TX USA; ^3^ Flow Cytometry Core Facility UT Health San Antonio San Antonio TX USA; ^4^ Department of Cell Systems and Anatomy UT Health San Antonio San Antonio TX USA; ^5^ Barshop Institute for Longevity and Aging Studies UT Health San Antonio San Antonio TX USA; ^6^ Department of Pathology and Laboratory Medicine Perelman School of Medicine University of Pennsylvania Philadelphia PA USA; ^7^ Department of Cellular and Integrative Physiology UT Health San Antonio San Antonio TX USA; ^8^ Department of Pharmacology UT Health San Antonio San Antonio TX USA; ^9^ Research Service South Texas Veterans Health Care System San Antonio TX USA

**Keywords:** age‐associated health deficit, Aging, hematopoietic stem cell transplantation, longevity, mobilization‐based conditioning, mouse

## Abstract

Mammalian aging is associated with reduced tissue regeneration and loss of physiological integrity. With age, stem cells diminish in their ability to regenerate adult tissues, likely contributing to age‐related morbidity. Thus, we replaced aged hematopoietic stem cells (HSCs) with young‐donor HSCs using a novel mobilization‐enabled hematopoietic stem cell transplantation (HSCT) technology as an alternative to the highly toxic conditioning regimens used in conventional HSCT. Using this approach, we are the first to report an increase in median lifespan (12%) and a decrease in overall mortality hazard (HR: 0.42, CI: 0.273–0.638) in aged mice following transplantation of young‐donor HSCs. The increase in longevity was accompanied by reductions of frailty measures and increases in food intake and body weight of aged recipients. Young‐donor HSCs not only preserved youthful function within the aged bone marrow stroma, but also at least partially ameliorated dysfunctional hematopoietic phenotypes of aged recipients. This compelling evidence that mammalian health and lifespan can be extended through stem cell therapy adds a new category to the very limited list of successful anti‐aging/life‐extending interventions. Our findings have implications for further development of stem cell therapies for increasing health and lifespan.

## Graphical abstract

Aged recipient mice underwent a mobilization‐based hematopoietic stem cell transplantation procedure followed by infusion of young‐donor stem cells. Young‐donor stem cells reverse age‐associated features and increase the health and longevity of aged recipients following aged hematopoietic stem cell replacement. Further, young‐donor cells maintained youthful features while under the influence of the aged bone marrow stroma.

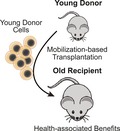

## 1 INTRODUCTION

Stem cells are critical to tissue regeneration and homeostasis during aging and disease (Signer & Morrison, 2013). As a hallmark of aging (Lopez‐Otin, Blasco, Partridge, Serrano, & Kroemer, 2013), stem cell dysfunction is critical to improving the quality of life for people with advanced age (Fontana, Kennedy, Longo, Seals, & Melov, 2014). Stem cell–based therapy holds considerable promise for treating aging‐related diseases(Ikehara & Li, 2014), with hematopoietic stem cells (HSCs) being the most widely used for stem cell therapies (Daley & Scadden, 2008). It is becoming increasingly clear that age‐related changes in the niche space can induce alterations in hematopoiesis, including myeloid lineage skewing (Guidi et al., 2017; Moerman, Teng, Lipschitz, & Lecka‐Czernik, 2004; Stier et al., 2005; Stolzing, Jones, McGonagle, & Scutt, 2008). However, extrinsic stimulation of HSCs with cytokines is highly dependent on intrinsic determinants (de Haan & Van Zant, 1997). To date, the “gold standard” measure of HSC functionality remains an in vivo repopulating assay to determine their ability to re‐establish lineage cell production in recipients during hematopoietic stem cell transplantation (HSCT; Kwarteng & Heinonen, 2016; Rossi et al., 2012). Unfortunately, conventional HSCT procedures require harsh cytotoxic conditioning—irradiation and/or chemotherapy—that alters HSC niches in the bone marrow, permanently damaging bone architecture (Green & Rubin, 2014; Naveiras et al., 2009). These limitations have confounded efforts to assess health‐associated benefits of HSC replacement and rejuvenation. Further, it has been impossible to determine the extent of which extrinsic factors drive age‐related decline of the hematopoietic system.

The majority of HSCs reside in specialized niches within the bone marrow, although some HSCs leave these niches and migrate into the blood, ~1%–5% of total HSCs each day (Bhattacharya et al., 2009). Mobilization of HSCs into the peripheral blood can be achieved through administration of G‐CSF (Teipel et al., 2015), an effect that is dramatically increased when G‐CSF is administered in combination with other mobilizers, such as AMD3100 (Pusic & DiPersio, 2010). This HSC mobilization strategy constitutes the basic mechanism underlying collection of peripheral blood donor stem cells in the clinic. Critically, this increased mobilization also creates temporarily empty niches in the bone marrow, opening a window of opportunity for donor cell engraftment. Here, we use a novel mobilization‐based HSCT procedure to investigate the health‐associated benefits of replacing HSCs from aged recipients with young‐donor HSCs. Additionally, we take advantage of the niche‐preserving properties of this mobilization‐based HSCT to investigate the influence of aged niche signaling upon a low percentage of young‐donor HSCs.

## 2 RESULTS

### 2.1 Long‐term donor chimerism was achievable following mobilization‐based conditioning

To reduce the adverse effects of cytotoxic conditioning agents, we developed a mobilization‐based conditioning procedure, eliminating the need for irradiation, followed by transplantation of donor HSCs. G‐CSF and AMD3100 (complementary mobilizing agents) were used to mobilize HSCs in ten‐week old mice. After peak mobilization (day 5), mice were transplanted with 2.0 × 10^6^ lineage‐negative, age‐matched, GFP^+^ bone marrow cells (Figure S1). A total of seven transplantation cycles were performed for each recipient, with donor chimerism (GFP^+^) increasing with every cycle of transplant, reaching ~90% at 1 month after the 7th cycle and stabilizing at ~77% by 4 months posttransplantation (Table 1).

**Table 1:** Mobilization‐based hematopoietic stem cell transplantation (HSCT)

Transplantation cycles (*n*)	Replacement result (%)	
	1 month post‐HSCT	4 months post‐HSCT
1	26.9 ± 4.9	25.9 ± 7.9
2	36.8 ± 5.3	36.6 ± 4.1
3	48.9 ± 4.0	42.2 ± 4.0
4	61.9 ± 5.5	50.0 ± 1.9
5	69.1 ± 4.6	56.8 ± 7.1
6	80.2 ± 2.6	68.1 ± 9.3
7	90.4 ± 2.9	76.6 ± 7.0

Note

*N* = 3/group. Variance = standard deviation of the mean. No animals were excluded from study.

### 2.2 Nontoxic hematopoietic reconstitution with young HSCs increases the longevity of aged recipient mice

**Figure 1:**
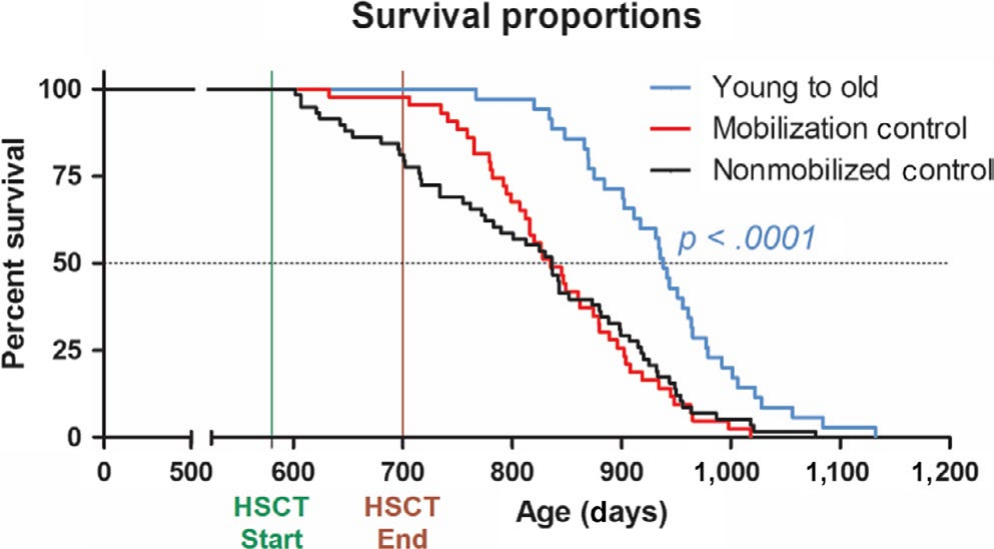
Young‐donor hematopoietic stem cells (HSCs) extend the lifespan of aged female recipients. Survival of wild‐type, female mice (19‐month‐old) receiving mobilization‐based conditioning followed by infusion of either young‐donor (2‐month‐old) HSCs (young to old, blue) or PBS (mobilized control, red) were compared with aged, wild‐type, female nonmobilized control (black). young to old, *N* = 42; mobilized control, *N* = 44; nonmobilized control, *N* = 58

**Figure 2:**
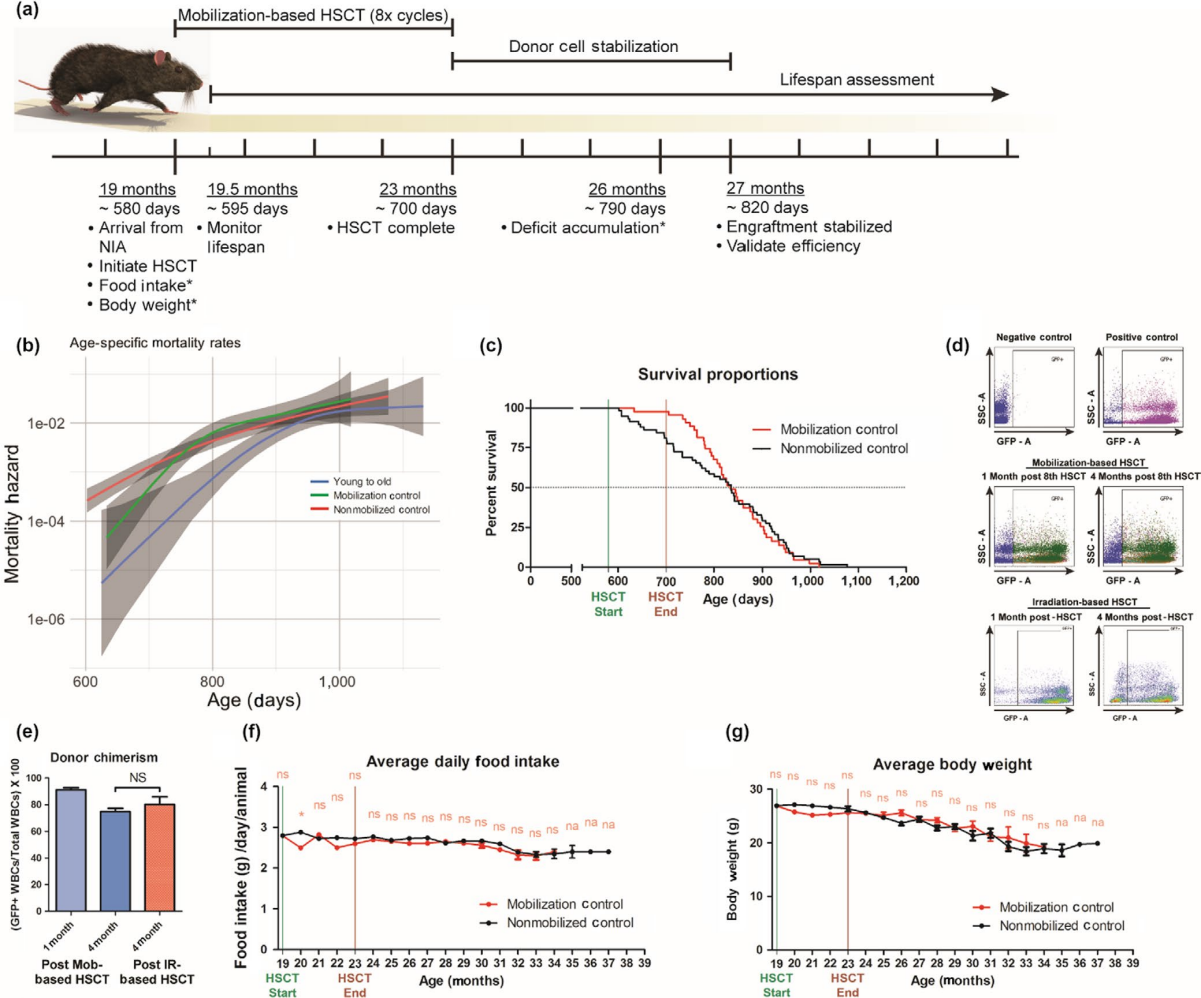
Mobilization‐based hematopoietic stem cell transplantation (HSCT) yields high donor chimerism without adverse effects. (a) Experimental design for aged recipients receiving eight mobilization‐based HSCT cycles followed by infusion of young‐donor hematopoietic stem cells (HSCs), *performed monthly. (b) Age‐specific mortality of aged recipient (young to old, blue), mobilized control (green), and nonmobilized control (red) mice, continuous confidence intervals highlighted (shaded regions). (c) Survival curves comparing mobilized (red) and nonmobilized control (Black) mice. (d) Representative flow dot plots, and (e) quantitative and statistical analysis of young‐donor WBC contributions in the peripheral blood of aged, wild‐type GFP^−^ mice 1—(Light blue, *N* = 9) and 4—(dark blue, *N* = 6) month postlast mobilization‐based HSCT (8 cycles) or young, wild‐type GFP‐ mice 4—(Red, *N* = 6) month postirradiation‐based HSCT. (f) Food intake, (g) body weight of controls were evaluated before, during, and post‐HSCT. Results are expressed as means of all surviving animals from each group for each time point. Error bars represent + 1 *SEM*. *p < .05. Unless otherwise noted, *N* = identical to lifespan assessment

Next, we used this novel HSCT method to investigate the impact of replacing aged (19‐month) HSCs with young‐donor (2‐month) HSCs. Figure 1 depicts the survival curves for preaged, female C57BL/6NIA mice, based on a total of 144 animals, recipients receiving a total of eight HSCT cycles (Figure 2a). In Table S2, the age of each group at 50% survival and maximal survival (mean age of the oldest 10%) is summarized, and the age of the oldest survivor is listed. Recipients of young‐donor HSCs yielded a hazard ratio (HR) for death of 0.33 (95% CI: 0.199–0.537, *p* < .0001) with regard to the 12.3% increase in median lifespan compared with mobilized control mice and an increase in median survival of 12.2% and HR of 0.42 (95% CI: 0.273–0.638, *p* < .0001*)* compared with non‐mobilized controls (Figure 1). These results confirm the increase in longevity that we previously observed in aged GFP^+^ recipients receiving GFP^‐^ young‐donor HSCs—17% increase in median lifespan and HR of 0.14 (95% CI, 0.054 to 0.348, *p* < .0001, Figure S2). Additionally, aged recipients exhibited a reduction in age‐specific mortality rates into late life (Figure 2b). Further, we found no significant differences between survival curves and age‐specific mortality rates of mobilized and non‐mobilized control groups, suggesting that mobilization‐based HSCT does not negatively affect the longevity of aged recipients (Figure 2c). Together, these data suggest that young‐donor HSCs are able to extend the longevity of aged recipients.

To monitor donor chimerism, aged‐matched (19‐month) female recipients received 2‐month‐old GFP^+^ young‐donor female HSCs in parallel with aged recipients of wild‐type young‐donor HSCs. Since only LT‐HSCs are capable of long‐term self‐renewal and continued contribution to hematopoiesis four months posttransplant (Dykstra et al., 2007), we obtained peripheral blood samples at both one and four months posttransplant to assess donor chimerism and long‐term donor cell reconstitution. Gating on GFP^+^ cells (Figure 2d), donor chimerism reached 91.2 ± 1.6% at 1 month after the last HSCT cycle and stabilized at 74.8 ± 2.6% by 4 months posttransplantation, comparable with donor chimerism observed in irradiation‐based HSCT recipients (80.3 ± 5.6%, Figure 2e).

### 2.3 Nontoxic hematopoietic reconstitution delays the accumulation of health‐associated deficits in aged recipient mice

**Figure 3:**
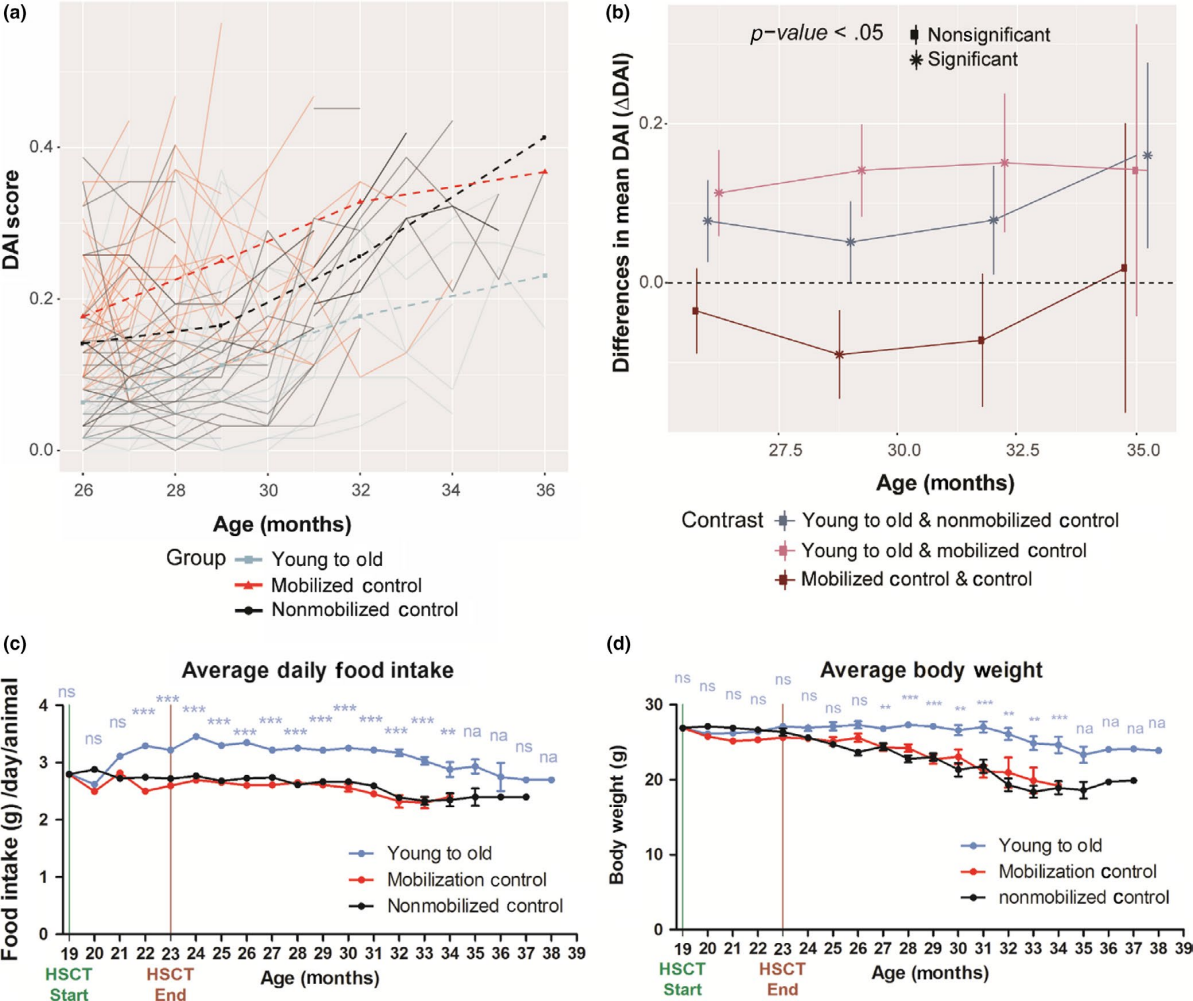
Young‐donor hematopoietic stem cells (HSCs) delay the accumulation of age‐related health deficits in aged female recipients. Long‐term effects of young‐donor (2‐month‐old) HSCs in aged (19‐month‐old), female mice—identical numbers (*N*) to lifespan assessment. (a) Spaghetti plot of the frailty index scores obtained from each surviving mouse from each group each month, FI scores were fit to a mixed model, and the overall FI score was generated for each group for each time point (26, 29, 32, and 35 months). (b) Differences in DAI scores between each group (i.e., young to old—nonmobilized control) were calculated and plotted for each time point (26, 29, 32, and 35 months). Error bars represent 95% confidence intervals, *p < .05. Attrition in sample size at 35 months of age was *N* = 5, 0, 3 for groups nonmobilized control, Mobilized control, Young to old, respectively. (c) Food intake and (d) body weight of recipients were measured before, during, and post‐HSCT. Results are expressed as means of all surviving animals from each group for each time point. Error bars represent ± 1 *SEM*. *p < .05, **p < .01, ***p < .001, ns = not significant, na = not analyzed

We monitored the progression and accumulation of age‐related health deficits of all surviving mice on a monthly basis (Table S2; Rockwood et al., 2017; Whitehead et al., 2014). Deficit accumulation index (DAI) ratios were generated by evaluating 31 potential age‐associated deficits under a noninvasive frailty index (FI; Howlett & Rockwood, 2013; Parks et al., 2012; Rockwood et al., 2017) and plotted as a function of age (Figure 3a), and mean DAI scores were generated for each group at each time point analyzed (Table S3). Strikingly, recipients of young‐donor HSCs accumulated fewer age‐related health deficits compared with nontransplanted controls at any given time during advanced life; the difference in health deficits ranged from −0.08 (95% CI: 0.026–0.129, *p* = .001) at 26 months to −0.30 (95% CI: 0.08–0.51, *p* = .004) after 38 months of age (Figure 3b). Moreover, the average daily food intake of recipients of young‐donor HSCs improved significantly, peaking at 3.45 ± 0.15 grams per day (Figure 3c). Given that these food intake measurements were obtained on a per‐cage basis, the differences in survival of HSC‐recipient versus control mice brings up potential cage effects that might influence eating behaviors, especially at later ages. Additionally, recipients of young‐donor HSCs maintained body weight later into advanced age compared with nontransplanted controls (Figure 3d), consistent with previous NIA‐derived cohorts (Turturro et al., 1999). Most time points showed no statistically significant differences between mobilized and nonmobilized controls, except at 29‐month nonmobilized controls had a −0.09 (*p* = .048) lower DAI score than mobilized controls. Food intake and body weight of mobilized controls declined similarly compared with nonmobilized controls (Figure 2f–g), demonstrating a significant reduction in procedure‐related adverse effects (Duran‐Struuck & Dysko, 2009).

### 2.4 Replacement of aged HSCs with young‐donor cells reverses age‐associated lineage skewing in aged recipients

**Figure 4:**
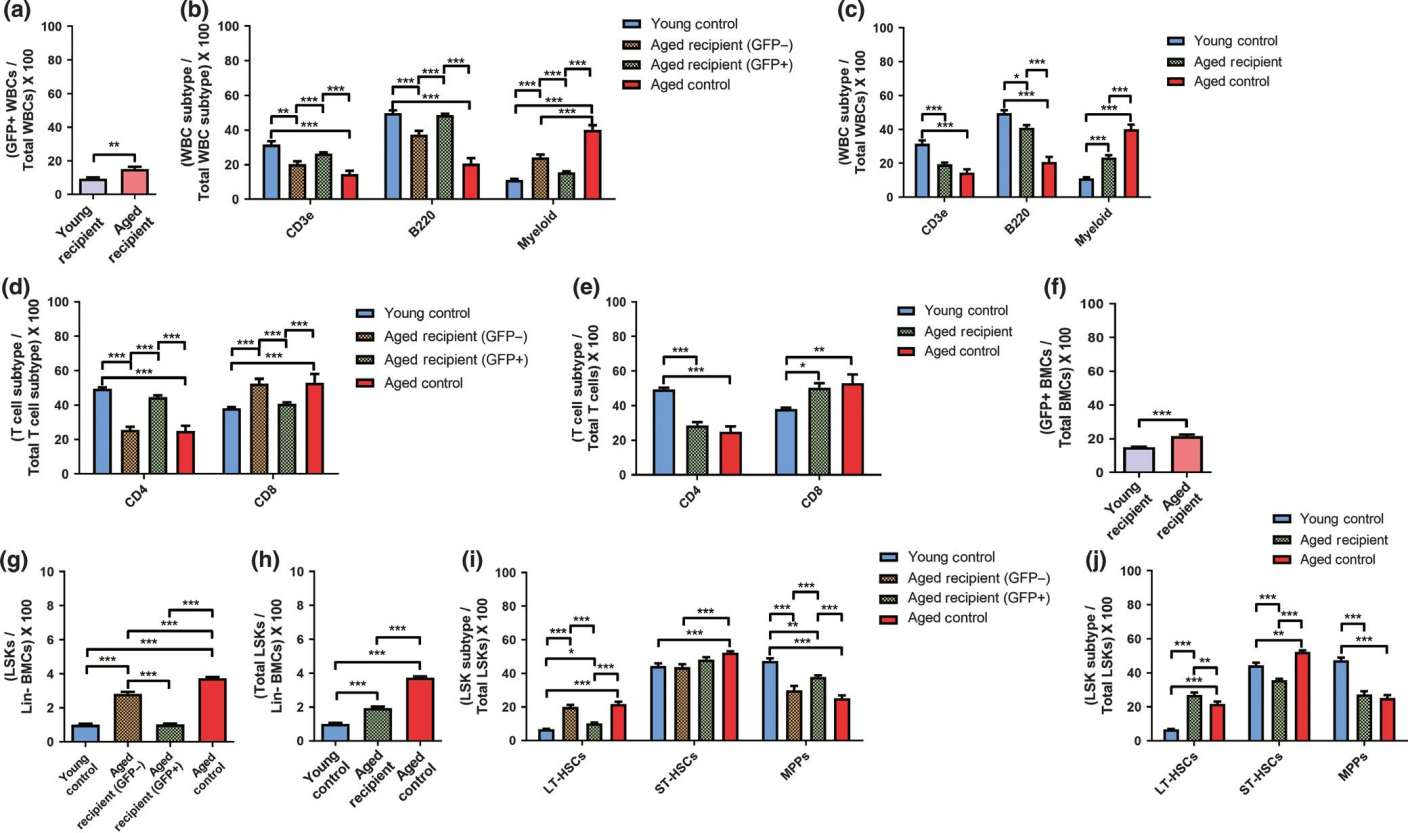
Young‐donor hematopoietic stem cells (HSCs) preserve youthful lineage phenotypes, while partially ameliorating age‐associated lineage phenotypes within the aged stroma of female recipients. Young‐donor (2‐month‐old) GFP^+^ WBC contributions within (a) peripheral blood and (f) bone marrow. (b, c) Quantitative and statistical analysis of the distribution of T cell, B cell, and myeloid cell frequencies within the peripheral blood and cell fractions of young control (*N* = 5), aged control (*N* = 5), and aged recipients (*N* = 20) mice 4 months posttransplant. (d, e) Distribution of CD4^+^ and CD8^+^ T‐cell frequencies. (g, h) Distributions of LSK cells within the bone marrow 4 months posttransplant. (i, j) Distributions of LT‐HSCs, ST‐HSCs, and MPP cell frequencies. Cell fractions were segregated based on endogenous (GFP^−^) and donor‐derived (GFP^+^) cell frequencies. *p < .05; **p < .01; ***p < .001; ordinates indicate means ± 1 *SEM*. Identical numbers (N) analyzed for each parameter

Age‐associated lineage skewing has been described by observing increased contributions of myeloid cell lineages in the peripheral blood at the expense of lymphoid cell lineages, both upon transplantation and steady state (Benz et al., 2012; Dykstra et al., 2007; Franceschi et al., 2007; Kovtonyuk, Fritsch, Feng, Manz, & Takizawa, 2016; Makinodan, 1998). Thus, we investigated whether young‐donor HSCs preserved youthful phenotypes within the aged stroma (Figure S3a). Young (2‐month‐old) and aged (19‐month‐old) recipients (all female) of a single mobilization‐based HSCT cycle were assessed for GFP^+^ young‐donor HSCs contribution in the peripheral blood (see Figure S4 for gating strategy and representative flow plots). Four months post‐HSCT, donor chimerism stabilized at 9.4 ± 0.8% (young recipients, 6 months old) and 15.2 ± 1.4% (aged recipients, 23 months, Figure 4a). Remarkably, white blood cell populations (WBCs) derived from young‐donor HSCs within aged recipients (GFP^+^) retained their youthful phenotypic distribution (Figure 4b; Table S4; Rossi et al., 2005). Limited replacement with young‐donor HSCs partially ameliorated the overall aged phenotypic distribution within aged recipients (Figure 4b, c). In fact, young‐donor HSCs within aged recipients (GFP^+^) gave rise to ratios between CD4^+^ helper and CD8^+^ cytotoxic T cells comparable with those observed in young recipients and controls (Figure 4d, e), suggesting that young‐donor HSCs preserve a youthful proliferative milieu within the aged stroma. Further, we observed no significant differences within the peripheral blood of mice receiving mobilization factors followed by sham transplants (Fig. S3b–i), despite the known short‐term increase in myeloid cell proliferation associated with G‐CSF (Knudsen et al., 2011).

### 2.5 Young‐donor cells maintain a youthful distribution of LSK cell subtypes in aged recipients

Next, we investigated young‐donor HSC expansion into LSK (Lin^‐^, Sca‐1^+^, c‐Kit^+^) cells within aged recipients (summarized in Table S5; Dykstra et al., 2007). Four months post‐HSCT, donor chimerism was 15.0 ± 0.4% and 21.5 ± 0.9% of lineage‐negative bone marrow cell contribution within young and aged recipients (6‐ and 23‐month‐old at the time of analysis), respectively (Figure 4f; see Fig. S5 for gating strategy and representative flow plots). Surprisingly, LSK cell expansion from young‐donor HSCs within aged recipients (GFP^+^) was identical to that in young controls (Figure 4g), resulting in a significant decrease in overall LSK cell frequencies within aged recipients (Figure 4h). Additionally, LSK subtype frequencies derived from young‐donor HSCs within aged recipients (GFP^+^) retained their youthful phenotypic distribution (Figure 4i), although the limited donor HSC contribution did not significantly reverse the aged phenotypic distribution within aged recipients (Figure 4j), suggesting that young‐donor HSCs do not influence aged HSCs through cell nonautonomous traits. Further, no significant differences were found in mice receiving mobilization factors followed by sham transplants (Fig. S3j–p), despite potential myeloid cell proliferation (Knudsen et al., 2011). Together, these data suggest that actively proliferating aged HSCs were replaced with young‐donor HSCs and that young‐donor HSCs maintain a youthful phenotypic distribution among LSK subtypes following transplantation into aged recipients.

## 3 DISCUSSION

Importantly, this study includes the first successful HSCT, in which severe adverse effects such as rapid declines in body weight (Duran‐Struuck & Dysko, 2009; Iestra, Fibbe, Zwinderman, Staveren, & Kromhout, 2002) and reduced survival (Guest, Ilic, Scrable, & Sell, 2015) were not observed. Recipients undergoing this mobilization‐based HSCT procedure required no additional care—e.g., antibiotics, acidic water, or frequent cage changes—to prevent HSCT‐related mortality (Duran‐Struuck & Dysko, 2009). In an ongoing study, toxicity profiles are being compared for this method versus conventional HSCT procedures (data not shown). Others are designing transplantation regimens that limit toxicity by eliminating the use of irradiation or chemotherapeutic drugs, however these methods require depletion of endogenous HSCs (Chhabra et al., 2016; Palchaudhuri et al., 2016). In the current studies, we observed nonsignificant differences within all health span parameters investigated or within any cell lineage investigated in the peripheral blood or bone marrow in mice receiving mobilization factors followed by sham transplants, providing strong evidence of a lack of long‐term adverse effects, despite potential myeloid cell proliferation (Knudsen et al., 2011).

To the best of our knowledge, this study is the first to achieve significant extension of the healthy lifespan of mice using a cell‐based therapy approach. This was evidenced in aged female recipients (19‐month‐old) of young‐donor (2‐month‐old) HSCs by extension of lifespan, reduction in age‐related health deficits, and increases in food intake and body weight. One limitation was that all mice used in this study were female. Ideally, we would have included equal‐sized cohorts of both male and female mice; however, to achieve statistical power, we needed a minimum of 30 aged mice. We chose to do the studies in females because some interventions have been shown to be applicable only to males (Austad & Bartke, 2015). We therefore reasoned that an effect on lifespan shown in females would be a more robust finding than one shown in males. Furthermore, our study design might ideally have incorporated an additional experimental condition, namely transplantation of aged donor cells into aged recipients to investigate the influx of transplanted HSCs and their influence on lifespan. However, the HSCT protocol requires harvesting HSCs at a ratio of 2:1 (donors to recipients) over the 8 transplantation cycles for each recipient, totaling 16 donor mice per recipient, which was not possible due to strict limitations in the number of aged mice available from the NIA resource (20 animals/ month). We do not believe that the study is compromised by this limitation in aged donor HSC availability, since there are clear differences between transplanting young versus aged HSCs, including shorter host lifespans (Guest et al., 2015) and increased myeloid lineage skewing due to “defective” HSCs (Lee, Yoon, Choi, & Jung, 2019).

**Figure 5:**
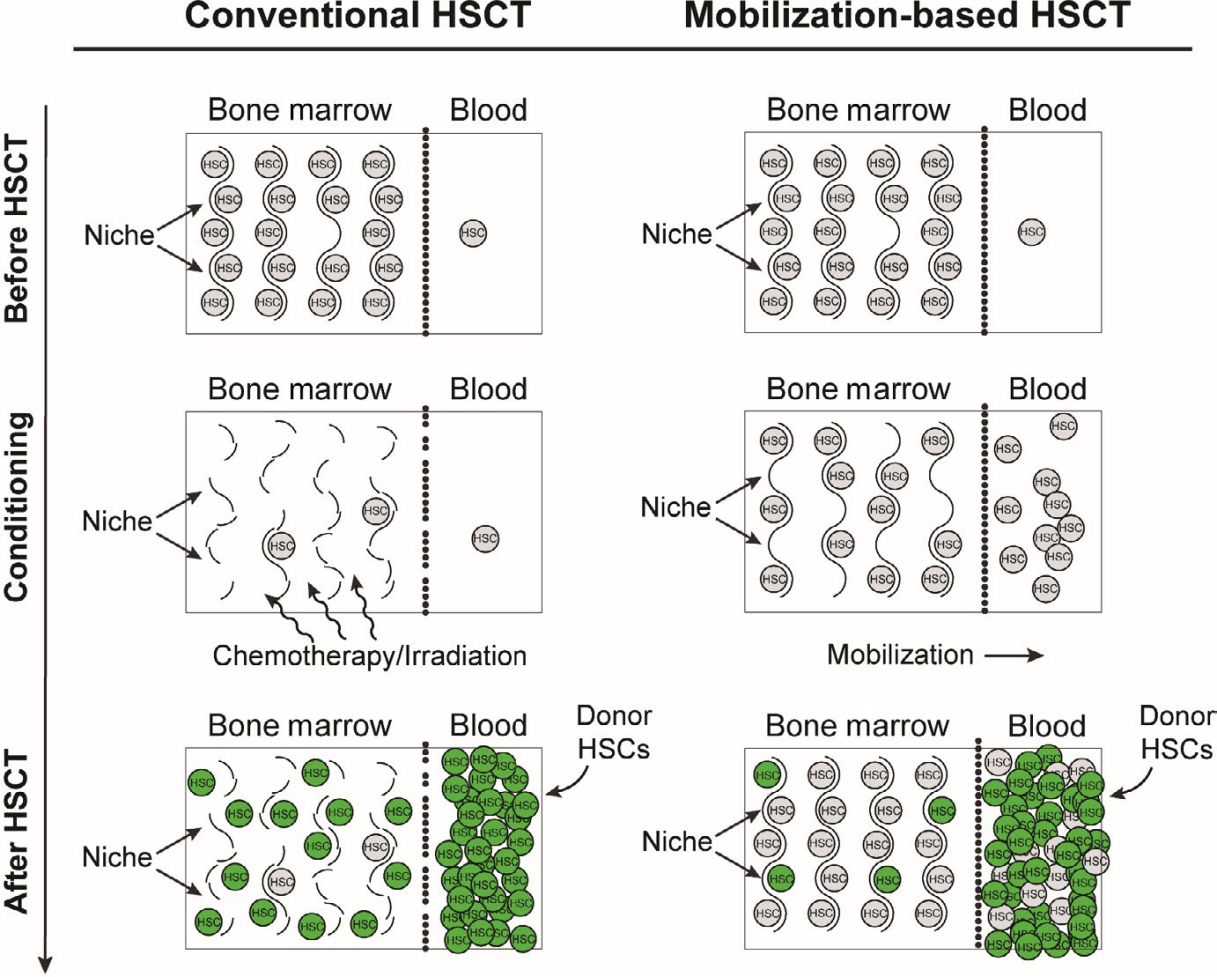
Schematic illustration comparing mechanisms between conventional and mobilization‐based hematopoietic stem cell transplantation (HSCT). First column depicts conventional HSCT following irradiation or chemotherapeutic conditioning, allowing noncompetitive engraftment of donor hematopoietic stem cells (HSCs), but significant damage to the stroma. Second column depicts HSCT following mobilization‐based conditioning, allowing infusion of superior donor HSC populations to out‐compete endogenous cells for limited niche spaces, while preserving the stroma

We speculate that these health‐associated benefits are a consequence of replacing actively proliferating myeloid‐biased HSCs known to accumulate in aged mice (reviewed in (Kovtonyuk et al., 2016)), subsequently decreasing their contributions to LSK frequencies and myeloid cells in the peripheral blood and thus relieving symptoms of immunosenescence. Lineage analyses of young‐donor cells revealed sustained youthful phenotypic distributions within the peripheral blood of aged recipients. We suspect that young donor‐derived immune cells also retain youthful function in addition to increased population. Declines in naïve T‐cell production coupled with clonal expansion of memory and effector T cells with age leads to decreased immune defense and autoimmunity (Dorshkind, Montecino‐Rodriguez, & Signer, 2009). It would be interesting to examine B cell subsets, since B‐cell diversity and affinity declines with age (Han et al., 2003). Although we did not investigate directly, donor‐derived red blood cells and platelets may be resistant to age‐related anemia (Price, Mehra, Holmes, & Schrier, 2011) or a prothrombotic state (Le & Lordkipanidze, 2019). Interestingly, a recent report demonstrated that young‐donor HSCs are capable of preserving learning and memory in aged mice following HSCT (Das et al., 2019), indicating potential influences of cell nonautonomous mechanisms from young‐donor HSCs. Others have reported on HSC rejuvenation achieved through pharmacological (Florian et al., 2012, p.42; Florian et al., 2013; Chen, Liu, Liu, & Zheng, 2009; Chang et al., 2016), gene manipulative (Brown et al., 2013; Satoh et al., 2013; Wang et al., 2016), and calorie restrictive (Cheng et al., 2014) strategies. Here, we used syngeneic, young‐donor HSCs to demonstrate aged HSC rejuvenation. It would be interesting to investigate whether transplantation of aged donor HSCs following an ex vivo rejuvenation program would yield similar findings. Another potential strategy would include HSC replacement using HLA^−^/^−^ modified embryonic stem cells as a universal supply of youthful HSCs (Lanza, Russell, & Nagy, 2019). Lastly, induced pluripotent stem cells may be another potential source of HSCs; however, these cells have only been shown to have youthful properties epigentically (Lo et al., 2017). In summary, our work paves the way for investigating HSC‐niche interactions using a mobilization‐based HSCT (Figure 5), which may become a safe and effective delivery platform for stored and/or rejuvenated HSCs.

## 4 EXPERIMENTAL PROCEDURES

### 4.1 Mice

All animal procedures were performed in accordance with National Institutes of Health (NIH) guidelines and were approved by The Institutional Animal Care and Use Committee of The University of Texas Health Science Center at San Antonio (UTHSCSA). Colony founders (C57BL/6J, GFP^‐^ mice) were obtained from the National Institute on Aging (NIA, Bethesda, MD), having originated from the Charles River Colony. Colony founders (C57BL/6J, GFP^+^) were obtained from our own stock of C57BL/6J mice (C57BL/6‐Tg (CAG‐EGFP) 131Osb/LeySopJ) from The Jackson Laboratory. Donor mice were housed and bred at the UTHSCSA Laboratory Animal Resources facility under standard conditions: 12‐hr light/dark cycle, 20–22°C, ad libitum access to food (Purina Lab Chow) and water in ventilated racks with plastic housing cages lined with chipped or shaved wood bedding. Water was changed and cages refreshed weekly. Breeding of female littermates occurred long enough to meet donor cell requirements at a 2:1 ratio of donor to recipient mice, per transplant cycle (i.e., 20 donor mice for every 10 recipient mice, per transplant cycle). All mouse procedures are summarized in Figure 2a and S4a.

### 4.2 HSC mobilization‐based conditioning

Two‐ or 19‐month‐old C57BL/6NIA female mice were obtained through the NIA on a monthly basis and randomly assigned to their respective groups. HSCs were mobilized by administration of G‐CSF (Neupogen^®^, Filgrastim, Amgen) by intra‐peritoneal injections (125 µg/kg) every 12 hr for four consecutive days prior to transplantation. AMD3100 (Mozobil, Genzyme, Cambridge, MA) was administered (5 mg/kg) via subcutaneous injection 14 hr after the last dose of G‐CSF and 1 hr prior to HSC transplantation. Lineage‐negative donor cells (5.0 × 10^6^) from eight‐week old GFP^∓^ mice were transplanted into each G‐CSF/AMD3100‐treated recipient mouse *via* tail vein injection. For initial transplants, this procedure was repeated once every two weeks for a number of cycles corresponding to individual group numbers (i.e., group 1 received one transplant, group 2 received two transplant cycles, and so on, with group 7 receiving seven transplant cycles). For longevity studies, all recipients received a total eight transplants. For peripheral blood and bone marrow analyses, recipients received only one transplant. HSC transplant efficacy was assessed by determination of percentage of GFP‐positive versus. total WBCs in peripheral blood by flow cytometry (BD FACSCalibur System, BD Bioscience) at 1 and 4 months after the last transplantation cycle.

### 4.3 Irradiation‐based conditioning

For the chimerism comparison study only (Figure 2e), recipient mice were given 1,050 centigray (cGy, ^123^Cs γ‐rays) of total body irradiation (~80 cGy/min). Eight‐week old GFP^+^ lineage‐negative donor cells (5.0 × 10^6^) were transplanted into each irradiated recipient mouse via tail vein injection. Gentamicin at a final concentration of 1.0 mg/ml was added to drinking water starting one week prior to irradiation and continuing until four weeks posttransplant. Cages were changed every other day. Overall health of irradiated recipients was monitored twice daily for extreme weight loss and poor body condition score. Animals exhibiting poor signs of health were removed from the study.

### 4.4 Donor cell collection

All donor mice used during cell collection were sex‐matched (female) and genotype‐matched (NIA‐derived) with recipients. Young, female, GFP^+^ donor mice (8–10 weeks old) were obtained from our own colony of female C57BL/6J mice established with animals obtained originally from The Jackson Laboratory. Young, female, GFP^‐^ donor mice (8–10 weeks old) were bred from colony founders obtained originally from the NIA. On the day of transplantation, donors were euthanized *via* cervical dislocation before collecting bone marrow cells by removing and flushing tibias, femurs, humeri, and hip bones with Iscove's Modified Dulbecco's Media (IMDM) containing 0.5% heparin. After red blood cell lysis and centrifugation, lineage‐negative cells were isolated using the Lineage Cell Depletion kit (Miltenyi Biotec Inc.) according to the manufacturer's protocol.

### 4.5 Longevity assessment

Longevity assessment was initiated two weeks after arrival at UTHSCSA from the NIA, to remove any animals that did not handle the acute stress of transportation or acclimate to the new environment. Upon arrival, 150 animals were separated randomly into one of four groups (maximum of five animals per cage). Once chosen, animals remained with the same cage‐mates, and no others, until end of life. Subjects removed from the study were those that did not survive past two weeks upon arrival from the NIA. Subjects censored were those that experienced experiment‐related mortality. To determine the time and type of death, mice were inspected at least twice daily. If aged mice appeared to be too weak to obtain food, a mush of ground pellets and water was placed on the cage bottom so that they did not succumb to dehydration/starvation. Moribund mice were euthanized if judged that they would not survive past another 48 hr. A mouse was considered severely moribund if it exhibited more than one of the following six clinical signs: inability to eat or drink; abnormally low body temperature; severe lethargy (reluctance to move when gently prodded with forceps); severe balance or gait disturbance; rapid weight loss for a week or more; an ulcerated or bleeding tumor. The age at which a moribund mouse was euthanized was taken as the best available estimate of its natural lifespan. A total of eight animals were censored from this study (seven transplanted, one mobilized control) as a result of procedure‐associated error during administration of cells or saline. Additionally, a total of six animals were removed from this study (three transplanted, one mobilized control, two nonmobilized controls) as a result of failure to acclimate to housing conditions. Kaplan–Meier analysis was used to generate survival curves to assess median and overall lifespans. Survival curves were compared using the log‐rank test to generate hazard ratios between the groups.

### 4.6 Age‐specific mortality

The instantaneous rate of mortality at each age was computed using a piecewise polynomial B‐spline hazard model assuming a Poisson distribution (Lambert & Eilers, 2005) using the *bshazard* package (Rebora, Salim, & Reilly, 2014).

### 4.7 Quantification of age‐related health deficits

Starting at 26 months of age, mice from each group were evaluated under a noninvasive frailty index (FI) based on the clinical assessment of 31 potential deficits, as previously described (Clegg, Young, Iliffe, Rikkert, & Rockwood, 2013; Howlett & Rockwood, 2013; Parks et al., 2012; Whitehead et al., 2014). Clinical assessments were performed between 10 a.m. and 2 p.m. at monthly intervals. The rater was blinded to the group from which the animals derived. A second rater assessed a subset of randomly selected mice and there were compared with initial assessments to maximize inter‐rater reliability. Assessments with a >10% difference in DAI scores between raters were re‐scored. Mice were placed individually in a fresh cage under a sterile flow hood in a procedure room designed for behavioral testing. The room, located on a quiet hall in the UTHSCSA Laboratory Animal Resources facility, had no other occupants (mice or humans). Clinical assessment included evaluation of the integument, the musculoskeletal system, the vestibulocochlear/auditory systems, the ocular and nasal systems, the digestive system, the urogenital system, and the respiratory system, as well as signs of discomfort. Body weight, an additional means to assess frailty, was performed separately. The hearing test used a clicker of the type used to train dogs. The deficit accumulation index (DAI) score was computed using a deficit rating scale. For each parameter, a score of 0 was given if there was no sign of a deficit, a score of 0.5 denoted a mild deficit, and a score of 1 indicated a severe deficit. The DAI scores for each of the 31 items on the checklist were added, and the total was divided by the number of deficits measured to yield a DAI score between 0 (no deficits) and 1 (all possible deficits) for each animal. Demographic characteristics for each cohort were expressed using the mean DAI scores ± standard deviation. Differences in DAI scores over time among the groups were estimated using a mixed model with group by time interactions (see Statistics).

### 4.8 Food intake assessment

Starting at 19 months of age, the average food intake was calculated for each group of mice on a monthly basis until animals expired. Average food intake was measured by recording the initial total mass of food available per cage followed by measuring the mass of food remaining after twenty‐four hours. The difference in mass between the initial available food and food remaining after twenty‐four hours was divided by the total number of mice present per cage before averaging with mice from all cages measured within each group. The mass of food was measured with a CS200 Compact Scale (Ohaus). In addition, cages were inspected before each recording for food crumbs. Crumbs were cleared from cages during the initial food measurement. Crumbs found twenty‐four hours after the initial measurement were added to the total food available to ensure accurate recording of food intake. If a mouse expired within the twenty‐four hour window in which food was measured, the calculation was discarded and a new measurement was initiated with the new number of mice available.

### 4.9 Body mass assessment

Starting at 19 months of age, the average body mass was calculated for each group of mice on a monthly basis until animals expired. Body mass was measured by recording the mass of individual mice with a CS200 Compact Scale (Ohaus), and averaged for all mice within each group. Measurements were performed at approximately the same time each month.

### 4.10 Peripheral blood analysis

Blood samples (40 µl) were collected from mice *via* tail snipping into microfuge tubes containing 40 µl flow buffer (PBS + 2.0% FBS) and heparin at a 3:1 ratio by volume (Stock 1,000 USP units/ml). After red blood cell lysis (RBC lysis buffer, Sigma‐Aldrich), samples were incubated with Fc‐receptor blockers specific for mouse CD16/32 and the following antibodies (1:100 dilution) on ice for 20 min: Ghost Dye™ Violet 510, anti‐Cd45R/B220‐PE, anti‐Cd3e‐Pacific Blue, anti‐CD11b‐PerCP‐Cy5.5, anti‐Ly‐6G/Ly‐6C‐APC‐Cy7 (Gr‐1), anti‐CD4‐BV605, and anti‐CD8‐Alexa Fluor 647. Cells were centrifuged, re‐suspended, and filtered immediately before analysis in a BD LSRII flow cytometer (BD BioSciences).

### 4.11 Hematopoietic progenitor analysis

Whole bone marrow was collected by flushing tibias, femurs, humeri, and hip bones. After red blood cell lysis, lineage‐negative cells were isolated using the Lineage Cell Depletion kit (Miltenyi Biotec Inc.) according to the manufacturer's instructions. Lineage‐negative cells were stained on ice with Fc‐receptor blockers specific for mouse CD16/32 (BD Pharmingen™) and appropriate antibody cocktails to determine percentages of each progenitor compartment. LT‐HSC, ST‐HSC, and MPP cells were stained with anti‐Sca‐1‐V‐500‐ Ly 6A/E, anti‐c‐Kit‐BV421‐CD117, anti‐Flk‐2‐PE‐CF594‐CD135, and anti‐CD34‐Alexa‐Fluor 647 for 20 min. Cells were centrifuged, re‐suspended in flow buffer (PBS + 2.0% fetal bovine serum), and then passed through a 40 µm filter immediately before analysis in a BD LSRII flow cytometer (BD BioSciences).

### 4.12 Statistical analyses

The rationale for the numbers (*n*) of mice in each group to provide adequate power to obtain significant results for the survival study was based on preliminary survival data obtained in our laboratory (Fig. S3). Assuming that differences in survival curves of experimental and control groups would be determined within the same order, a minimum of 30 mice in each group was estimated to have a power of >99% with an effect size of 1.311 (α = 0.01). Differences in DAI scores over time among the groups were estimated using a mixed model with group by time interactions. The model had random intercepts and random slopes to account for the correlations within mice, as well as for mouse‐specific trajectories. Time was treated as a quadratic effect to accommodate curvilinear age‐related changes identified through model selection with the Akaike Information Criterion (AIC). Linear contrasts were used to estimate confidence intervals and test for significant differences at each time point. For these studies, statistical tests were two‐sided at significance level 0.05. These analyses were performed in the R environment for statistical computing 3.3.0 (R Core Team, 2016) within an accountable data analysis process (Gelfond, Goros, Hernandez, & Bokov, 2018). Statistical analyses of daily food intake and body mass assessment data were performed using GraphPad Prism 6.02 (GraphPad Software). All data are expressed as mean ± standard error of the mean. Multiple group comparisons were analyzed by two‐way ANOVA, followed by post hoc analyses using Bonferroni posttest or one‐way ANOVA, followed by Tukey's posttest. Differences among treatment groups were considered statistically significant at *p < .05, **p < .01, ***p < .001. For all other measures, significance was assigned using the Student's *t* test. Statistical analyses were performed by the UT Health San Antonio Nathan Shock Center Statistics Core.

## CONFLICT OF INTERESTS

The authors have declared that no additional conflict of interest exists.

## AUTHOR CONTRIBUTIONS

M.J.G., R.A.F., K.M.G., C.J.C, and S.L. designed research and analyzed data. J.A.G. and R.J.S. provided critical scientific, statistical, and technical insights. M.J.G. carried out all experimental work with the help of C.C., A.B., and G.G. S.L., J.F.N., and R.A.C. directed the project. M.G. wrote the manuscript with the help of J.A.G., Y.L., J.F.N., R.S., P.J.S., R.A.C., and S.L., R.A.C., C.C., and M.J.G. have filed a provisional patent application.

## ETHICAL APPROVAL

The procedures for all animal experiments were reviewed and approved by the Institutional Animal Care and Use Committee (IACUC) of the University of Texas Health San Antonio.

## Data Availability

All data associated with this study are present in the paper or Supplementary Materials.
