# Supplementary material for: Mobilization‐based transplantation of young‐donor hematopoietic stem cells extends lifespan in mice
Source: Aging Cell. 2020 Feb 3;19(3):e13110. doi: 10.1111/acel.13110 (PMC7059148; doi:10.1111/acel.13110)
Supplement: Supplementary file 1 [file ACEL-19-e13110-s001.docx]

**Supplementary Information**:


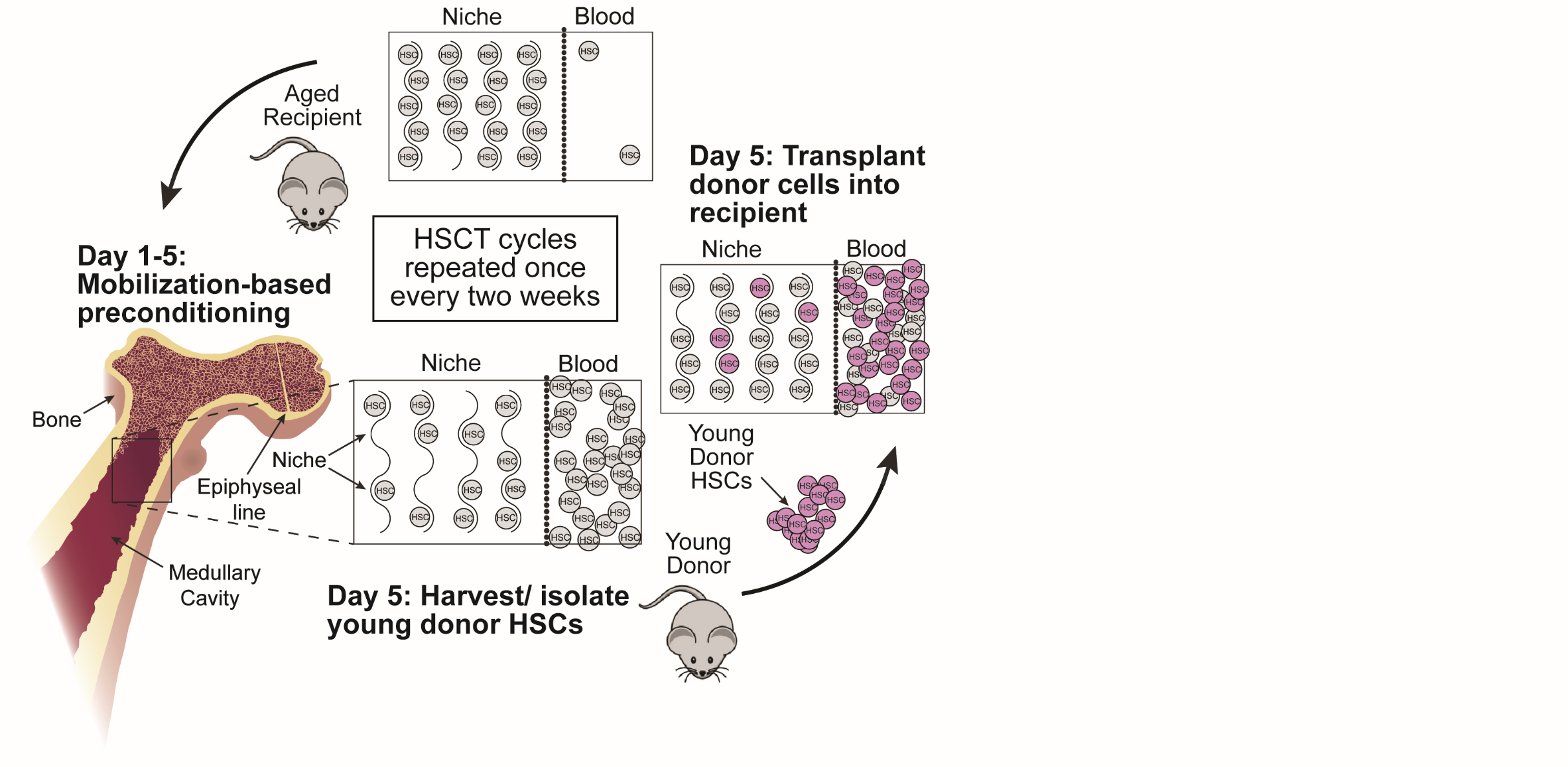


**Fig. S1. Schematic of the novel HSCT method.** Recipient mice received intra-peritoneal injections of G-CSF twice daily for 4 consecutive days, and on day 5 a single subcutaneous injection of AMD3100. One hour post AMD3100 injection, mice received 2x10^6^ lineage-negative donor cells *via* tail vein injection. This constitutes one full HSCT cycle. For the lifespan studies, each recipient mouse underwent 8 HSCT cycles over the course of 4 months.


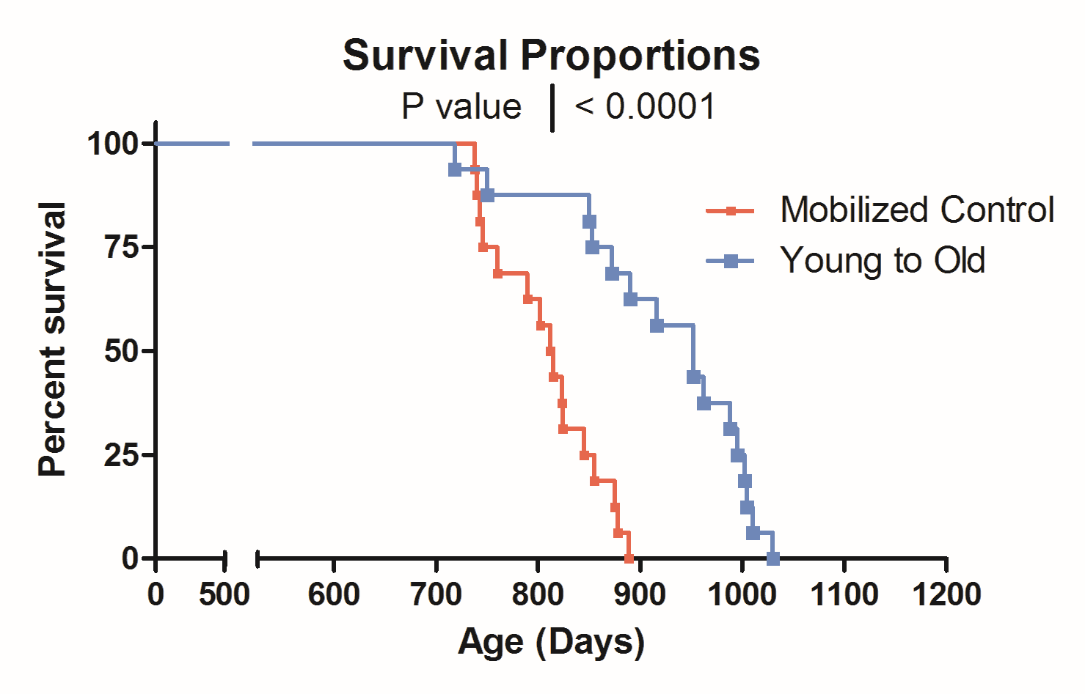


**Fig. S2. Survival analysis of C57BL/6 female GFP^+^ recipient mice following bone marrow transplants.** Eight mobilization-based HSCT cycles were performed on 19 month-old female GFP^+^ C57BL6/J mice which received either young (2 month-old) female donor HSCs (GFP^-^) or saline. All mice were monitored for overall longevity. Log-ranked statistical analyses revealed an approximate 17% increase in median lifespan and a hazard ratio of 0.14 in recipients receiving young donor cells.


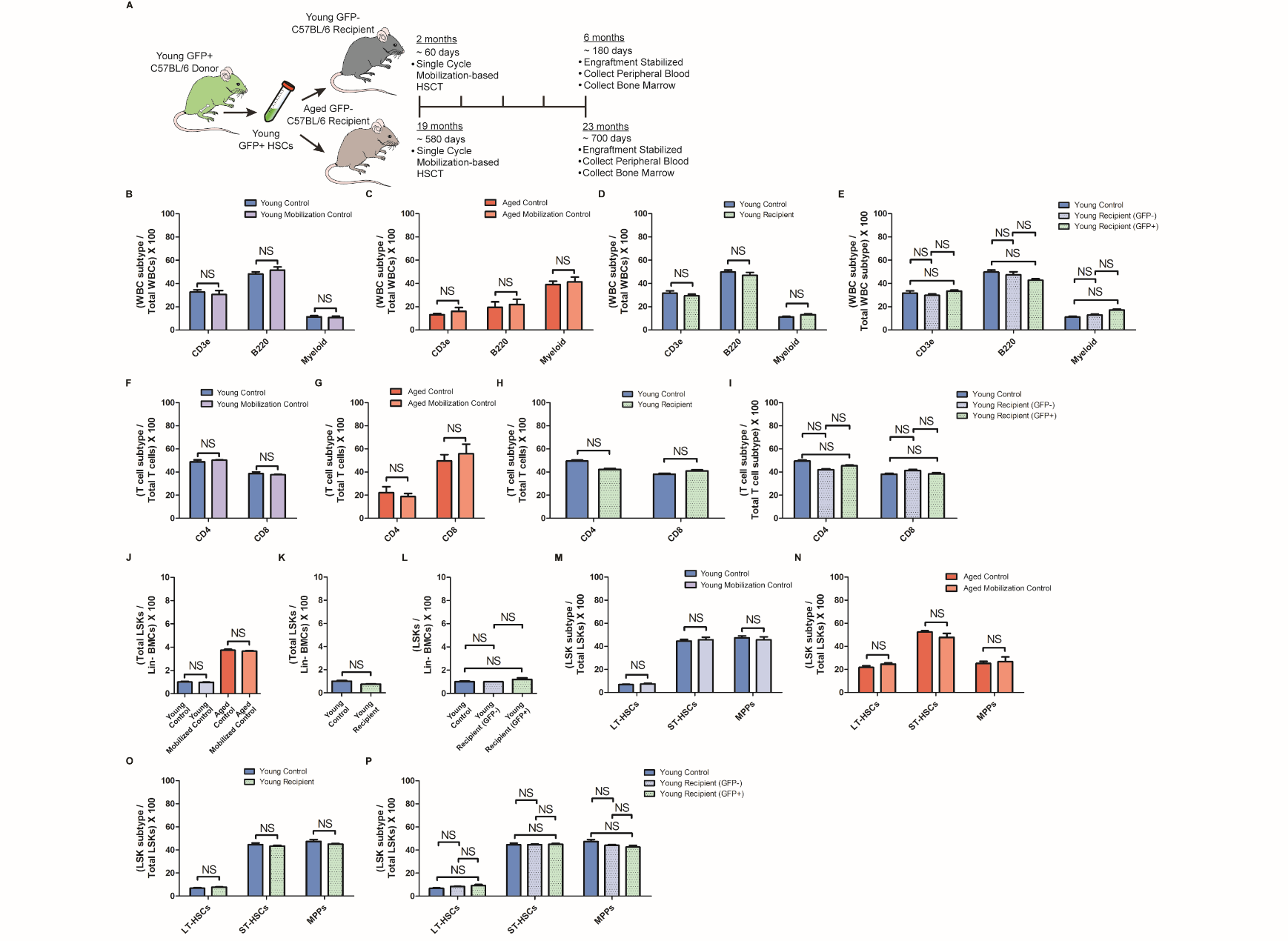


**Fig. S3. Mobilization-based conditioning does not alter the frequencies of peripheral blood or bone marrow cell lineages. (A)** Experimental design. **(B-E)** Quantitative and statistical analysis of the distribution of T cell, B cell, and myeloid cell frequencies within the peripheral blood of young mobilized (N = 5) and non-mobilized (N = 5) controls, aged mobilized (N = 5) and non-mobilized (N = 5) controls, and young recipients of young-donor HSCs (N = 15) 4 months post-HSCT or mobilization. **(F-I)** Distributions of CD4^+^ and CD8^+^ T cell frequencies within T cell frequencies. **(J-L)** Distributions of LSK cell frequencies within the bone marrow. **(M–P)** Distribution of LT-HSC, ST-HSC, and MPP cell frequencies among LSK cells 4 months post-HSCT or mobilization. NS = not significant; ordinates indicate means ± 1 SEM.


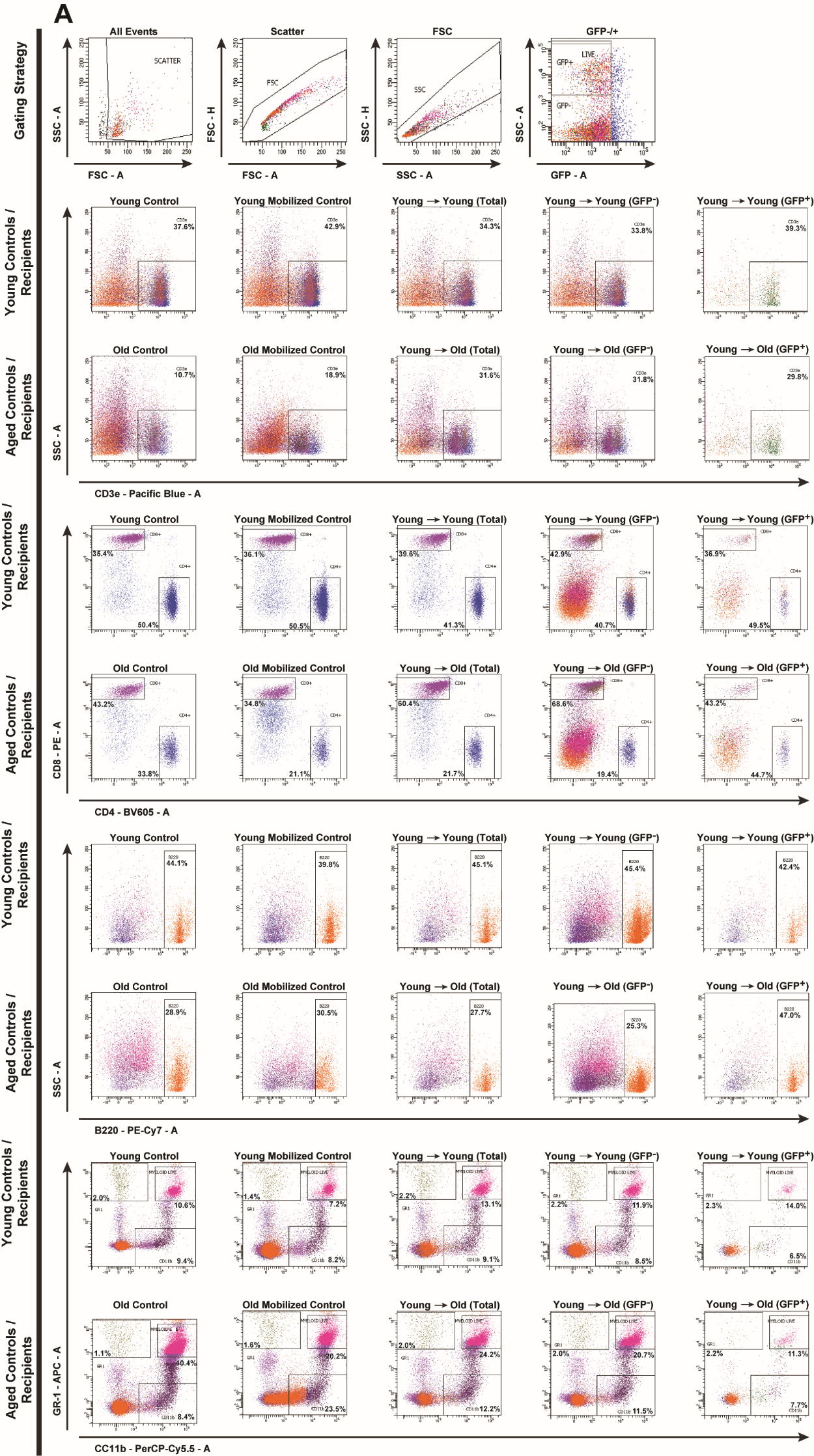


**Fig. S4. Representative flow dot plots of peripheral blood samples.**  General gating strategy used to determine live, GFP^-^, and GFP^+^ cell frequencies of T cell, B cell, myeloid (CD11b^+^, GR-1^+^), CD4^+^ and CD8^+^ cell frequencies, from peripheral blood samples from each group. Gated on either live, GFP^-^, GFP^+^, or CD3e^+^ (T cell) frequencies. Antibodies and fluorescent tags are shown below dot plots.


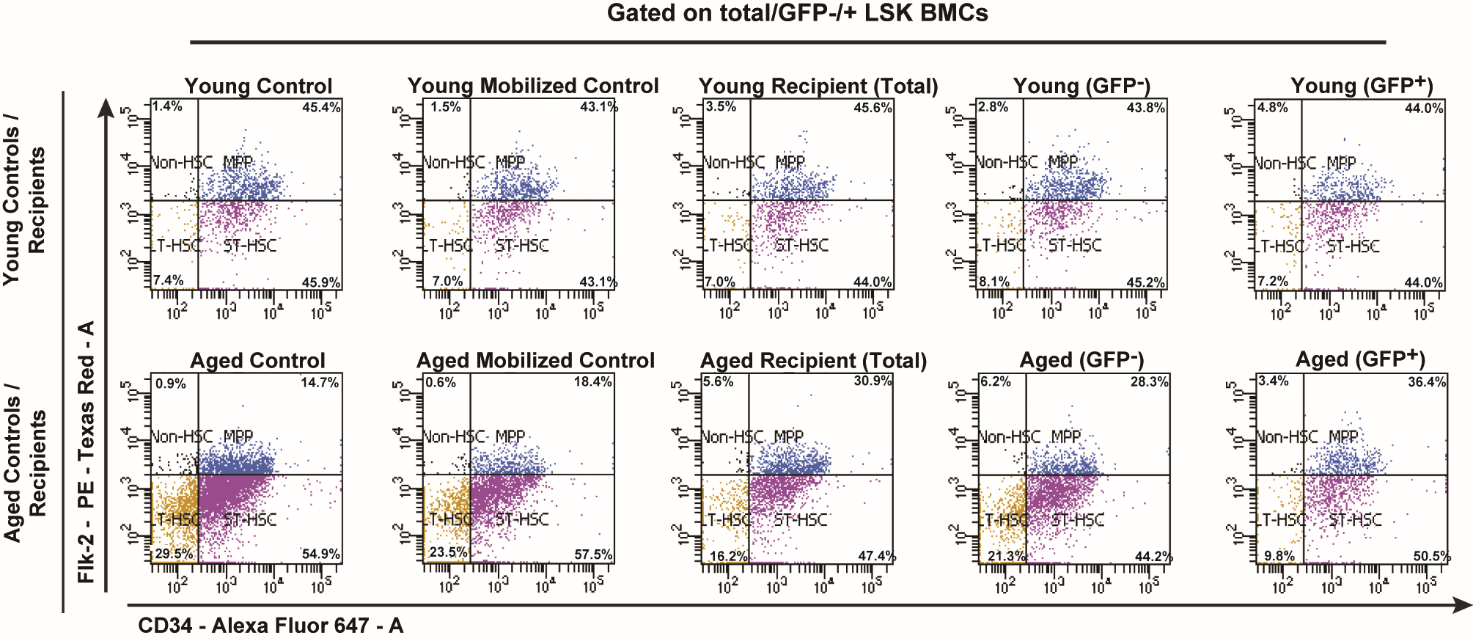


**Fig. S5. Representative flow dot plots of bone marrow samples.**  General gating strategy used to determine live, GFP^-^, and GFP^+^ LSK cell frequencies from bone marrow cells samples from each group. Gating strategy used to determine LT-HSCs (CD34^low/-^, Flk-2^-^), ST-HSCs (CD34^+^, Flk-2^-^), MPPs (CD34^+^, Flk-2^+^), and Non-HSC (CD34^-^, Flk-2^+^) cell frequencies. Antibodies and fluorescent tags are shown below dot plots.

| Table S1. Age at 50% survival^a^ and maximal^b^ lifespan | | | |
| --- | --- | --- | --- |
| Parameter | Y2O^e^ [days (months)] | MOB^f^ [days months)] | NON^g^ [days (months)] |
| 50% Survival | 938 (30.8) | 835 (27.4) | 836 (27.5) |
| Maximal Lifespan | 1064 (35.0) | 978 (32.1) | 1004 (33.0) |
| Oldest Survivor | 1132 (37.2) | 1018 (33.4) | 1077 (35.4) |
| Censored^c^ (#/total) | 7/42 | 1/44 | 0/58 |
| N^d^ | 35 | 43 | 58 |
| ^a^ Median survival (first week of 50% mortality)  ^b^ Mean age of oldest 10% from each group  ^c^ Censored due to procedure-associated death  ^d^ Total number of individuals not censored or removed from study per group  ^e^ Y2O = Young to Old  ^f^ MOB = Mobilized Control  ^g^ NON = Non-mobilized Control | | | |

| Table S2. Clinical signs of deterioration in Aging C57BL/6J Mice | |
| --- | --- |
| *Integument* |  |
| Loss of whiskers | Loss of vibrissae due to aging and/or whisker trimming |
| Coat condition: | Ruffled, ungroomed, and/or matted fur. Not smooth, sleek, and shiny |
| Alopecia | Hair loss due to age-related balding |
| *****Barbering | Hair loss due to extensive barbering |
| Loss of fur color | Change in fur color from black to grey or brown |
| Dermatitis | Open sores anywhere on the body due to Inflammation, over-grooming, barbering or scratching causing skin erosion. |
| *****Fight wounds | Wounds associated with fighting |
| *Physical/musculoskeletal* |  |
| Body condition score | Visual signs of muscle wasting or obesity based on the amount of flesh covering bony protuberances |
| Kyphosis | Exaggerated outward curvature of the lower cervical/thoracic vertebral column. Hunched back or posture |
| Tumors | Development of tumors or masses anywhere on the body |
| Distended abdomen | Enlarged abdomen due to tumor growth, organ enlargement, or intraperitoneal fluid accumulation |
| Tremor | Involuntary shaking at rest or during movement |
| Gait disorders | Lack of coordination in movement including hopping, wobbling, or uncoordinated gait, wide stance, circling, or weakness |
| Forelimb grip strength | A decline in forelimb grip strength |
| Tail stiffening | Tail appears stiff, even when animal is moving in the cage and does not wrap freely when stroked |
| *Vestibulocochlear/auditory* |  |
| Vestibular disturbance | Disruption in the ability to perceive motion and gravity, reflected in problems with balance, orientation, and acceleration |
| Hearing loss | Failure to respond to sudden sound indicative of hearing loss or impairment |
| *Ocular/nasal* |  |
| Cataracts | An opaque spot in the center of the eye or clouding of the lens |
| Corneal opacity | Development of white spots or cloudiness on the cornea. |
| Eye swelling/discharge | Swollen or bulging (exopthalmia) eye(s), abnormal secretions and/or crusting |
| Microphthalmia | Eye(s) are small and/or sunken |
| Vision loss | Vision loss: failure to reach toward ground when lowered by the tail |
| Menace reflex | Rapid eye blink and closure of the palpebral fissure in response to a non-tactile visual threat to the eye |
| Nasal discharge | Signs of abnormal discharge from the nares |
| *Digestive/urogenital* |  |
| Malocclusions | Teeth are uneven or overgrown, abnormal top/bottom tooth growth |
| Rectal prolapse | Protrusion of the rectum |
| Vaginal prolapse | Vagina or uterus protrudes through the vagina and vulva |
| Diarrhea | Feces on the walls of the home cage. Bedding adheres to feces in cage. Feces, blood, or bedding around the rectum |
| *Respiratory* |  |
| Breathing rate/depth | Difficulty breathing (dyspnea), pulmonary congestion (rales), and/or rapid breathing (tachypnea) |
| *Discomfort* |  |
| Piloerection | Involuntary bristling of the fur: sympathetic nervous system activation |
| Mouse grimace scale | Measure of pain/discomfort based on facial expression. Assessment of five facial features: orbital tightening, nose bulge, cheek bulge, ear position (drawn back), or whisker change (either backward or forward) |
| Table adapted from *Whitehead et al 2013.^20^*  *****Indicates assessments suggested by Dr. Strong and team | |

| Table S3. Differences in DAI score (ΔDAI) between groups at each time point | | | | | | |
| --- | --- | --- | --- | --- | --- | --- |
| Groups Contrasted | Age (Months) | Frailty Difference (ΔFI) | Standard Error | P Value | Lower CL (95%) | Upper CL (95%) |
| NON^a^ - Y2O^b^ | 26 | 0.0778 | 0.0216 | 0.0014 | 0.0265 | 0.1291 |
| NON - Y2O | 29 | 0.0513 | 0.0214 | 0.0482 | 0.0003 | 0.1023 |
| NON - Y2O | 32 | 0.0787 | 0.0284 | 0.0196 | 0.0106 | 0.1468 |
| NON - Y2O | 35 | 0.1600 | 0.0485 | 0.0046 | 0.0435 | 0.2766 |
| NON - Y2O | 38 | 0.2952 | 0.0896 | 0.0042 | 0.0810 | 0.5095 |
|  |  |  |  |  |  |  |
| NON – MOB^c^ | 26 | -0.0352 | 0.0225 | 0.2657 | -0.0887 | 0.0183 |
| NON - MOB | 29 | -0.0900 | 0.0234 | 0.0006 | -0.1456 | -0.0344 |
| NON - MOB | 32 | -0.0720 | 0.0349 | 0.1044 | -0.1555 | 0.0114 |
| NON - MOB | 35 | 0.0188 | 0.0764 | 0.9672 | -0.1629 | 0.2004 |
| NON - MOB | 38 | 0.1824 | 0.1530 | 0.4597 | -0.1799 | 0.5447 |
|  |  |  |  |  |  |  |
| MOB - Y2O | 26 | 0.1130 | 0.0227 | 0.0000 | 0.0589 | 0.1670 |
| MOB - Y2O | 29 | 0.1413 | 0.0244 | 0.0000 | 0.0833 | 0.1993 |
| MOB - Y2O | 32 | 0.1507 | 0.0363 | 0.0003 | 0.0639 | 0.2375 |
| MOB - Y2O | 35 | 0.1413 | 0.0769 | 0.1634 | -0.0420 | 0.3245 |
| MOB - Y2O | 38 | 0.1128 | 0.1518 | 0.7381 | -0.2471 | 0.4728 |
| ^a^ NON = Non-mobilized Control  ^b^ Y2O = Young to Old  ^c^ MOB = Mobilized Control | | | | | | |

| Table S4. Distribution of WBC subtypes within the peripheral blood | | | | | |
| --- | --- | --- | --- | --- | --- |
|  | WBC Frequency  (Mean ± SEM) | | | T-Cell Subset Frequency (Mean ± SEM) | |
| Group | T cell | B Cell | Myeloid | CD4 | CD8 |
| Young Control^a^ | 31.7 ± 1.9 | 49.8 ± 1.7 | 11.0 ± 0.7 | 49.5 ± 0.9 | 38.1 ± 0.8 |
| Young Mobilized Control^a^ | 28.6 ± 1.7 | 51.6 ± 2.7 | 10.8 ± 1.1 | 50.2 ± 0.5 | 37.7 ± 0.3 |
|  |  |  |  |  |  |
| Young Recipient (Total)^b^ | 29.4 ± 1.3 | 47.1 ± 2.5 | 13.2 ± 0.8 | 42.3 ± 0.8 | 41.0 ± 0.9 |
| Young Recipient (GFP^-^)^b^ | 29.7 ± 1.2 | 47.5 ± 2.6 | 12.7 ± 0.8 | 49.5 ± 0.9 | 41.3 ± 0.9 |
| Young Recipient (GFP^+^)^b^ | 33.3 ± 0.9 | 42.7 ± 1.2 | 17.1 ± 0.8 | 41.9 ± 0.9 | 38.4 ± 1.0 |
|  |  |  |  |  |  |
| Aged Recipient (Total)^c^ | 19.4 ± 0.9 | 41.0 ± 1.5 | 23.5 ± 1.4 | 28.7 ± 1.9 | 50.4 ± 2.6 |
| Aged Recipient (GFP^-^)^c^ | 20.3 ± 1.7 | 37.4 ± 2.3 | 24.1 ± 1.7 | 45.4 ± 0.7 | 52.4 ± 2.9 |
| Aged Recipient (GFP^+^)^c^ | 26.5 ± 0.8 | 48.7 ± 0.8 | 15.6 ± 0.6 | 25.6 ± 1.9 | 40.9 ± 0.8 |
|  |  |  |  |  |  |
| Aged Control^a^ | 14.7 ± 1.8 | 20.7 ± 3.1 | 40.2 ± 2.5 | 24.9 ± 3.2 | 53.0 ± 5.0 |
| Aged Mobilized Control^a^ | 15.9 ± 3.2 | 23.8 ± 5.8 | 41.3 ± 4.2 | 18.7 ± 2.7 | 55.8 ± 8.3 |
| ^a^ N = 5  ^b^ N = 15  ^c^ N = 20 | | | | | |

| Table S5. Distribution of LSK subtypes within the bone marrow | | | | |
| --- | --- | --- | --- | --- |
|  | LSK Frequency  (Mean ± SEM) | LSK subtype Frequency  (Mean ± SEM) | | |
| Group | LSKs | LT-HSCs | ST-HSCs | MPPs |
| Young Control^a^ | 1.0 ± 0.1 | 6.8 ± 0.4 | 44.5 ± 1.5 | 47.4 ± 1.7 |
| Young Mobilized Control^a^ | 1.0 ± 0.0 | 7.3 ± 0.6 | 45.7 ± 2.1 | 45.6 ± 2.6 |
|  |  |  |  |  |
| Young Recipient (Total)^b^ | 0.75 ± 0.0 | 7.7 ± 0.2 | 43.4 ± 0.6 | 45.0 ± 0.6 |
| Young Recipient (GFP^-^)^b^ | 1.0 ± 0.0 | 8.3 ± 0.3 | 44.5 ± 0.7 | 44.1 ± 0.6 |
| Young Recipient (GFP^+^)^b^ | 1.2 ± 0.1 | 9.2 ± 0.9 | 45.0 ± 0.8 | 42.6 ± 1.4 |
|  |  |  |  |  |
| Aged Recipient (Total)^c^ | 2.0 ± 0.1 | 27.1 ± 1.5 | 35.7 ± 0.8 | 27.3 ± 2.0 |
| Aged Recipient (GFP^-^)^c^ | 2.8 ± 0.1 | 20.1 ± 1.1 | 43.8 ± 1.7 | 30.0 ± 2.4 |
| Aged Recipient (GFP^+^)^c^ | 1.0 ± 0.0 | 10.2 ± 0.5 | 48.3 ± 1.4 | 37.8 ± 1.1 |
|  |  |  |  |  |
| Aged Control^a^ | 3.7 ± 0.1 | 21.7 ± 1.4 | 52.4 ± 0.9 | 25.1 ± 1.9 |
| Aged Mobilized Control^a^ | 3.7 ± 0.1 | 24.6 ± 1.1 | 47.7 ± 3.5 | 26.7 ± 4.1 |
| ^a^ N = 5  ^b^ N = 15  ^c^ N = 20 | | | | |
